# Electronic cigarette power affects count concentration and particle size distribution of vaping aerosol

**DOI:** 10.1371/journal.pone.0210147

**Published:** 2018-12-31

**Authors:** Evan L. Floyd, Lurdes Queimado, Jun Wang, James L. Regens, David L. Johnson

**Affiliations:** 1 Department of Occupational and Environmental Health, College of Public Health, University of Oklahoma Health Sciences Center, Oklahoma City, Oklahoma, United States of America; 2 Oklahoma Tobacco Research Center, Oklahoma City, Oklahoma, United States of America; 3 Department of Otorhinolaryngology, College of Medicine, University of Oklahoma Health Sciences Center, Oklahoma City, Oklahoma, United States of America; 4 OU Center for Intelligence and National Security, University of Oklahoma, Oklahoma City, Oklahoma, United States of America; Johns Hopkins University, UNITED STATES

## Abstract

**Introduction:**

Electronic cigarettes (EC) have evolved rapidly toward higher powered devices that produce more vaping aerosol and a more satisfying vaping experience. This research characterized the particle size distribution and estimated the mass concentration of vaping aerosols produced at power outputs spanning the operating range typical of second generation variable voltage EC devices.

**Methods:**

EC aerosol was characterized from a single coil atomizer powered by a variable voltage EC battery at the minimum and maximum dial settings (3.3, 11.2 Watts, W), and a lab controlled power supply (3–11.9 W). Aerosol particle size distribution was measured by a Scanning Mobility Particle Sizer and Aerodynamic Particle Sizer, spanning 16 nm to 19.8 μm. A mouth puff was simulated using a 100 mL glass syringe.

**Results:**

Consistent with prior studies, sub-micron EC aerosol size distributions were bimodal, with peaks at 40 and 200 nm, however a previously unreported third mode was observed at approximately 1000 nm. The ~1000 nm mode accounted for 7-20x the aerosol mass of the smaller modes. Increasing atomizer power decreased count concentration of particles <600 nm but increased particle count >600 nm. Particle mass distribution shifted toward micron sized particles with increasing power and increased the respirable fraction of aerosol, likely due to increased coagulation and condensation around nano-sized particles.

**Conclusions:**

Vaping power greatly affects EC aerosol count and mass distribution. Mouth puffed EC aerosol spans a much wider particle size range than previously reported, although the major portion of the mass is still well within the alveolar size range the larger particles will deposit within the oro-pharyngeal cavity at 2-3x greater efficiency than in alveoli. These observations have major clinical implications, as aerosol particle size distribution determines deposition sites along the respiratory tract. The results of this experiment stress the need for further research to inform the design, regulation and use of e-cigarette products.

## Introduction

E-cigarettes (ECs) have been described as a potentially disruptive technology that could greatly assist efforts to reduce tobacco-related morbidity and mortality and have been shown to deliver nicotine on par with tobacco cigarettes[1]. Although Abrams[1] has suggested that ECs could make the combusting of tobacco obsolete, he cautioned that additional research is needed to inform science-based policy. His cautionary note reflects the fact that currently there is a general lack of scientific data about EC effectiveness and safety. As a result, the US Food and Drug Administration (FDA) notes that “it is important to evaluate e-cigarettes based on their individual characteristics… to learn more about the potential benefits and drawbacks of the products”.[2]

Systematic evaluation of individual characteristics of commercially available EC devices is complicated by the rapid evolution of EC technology and designs, which is driven largely by the innovations of experienced users in the vaping community and highly adaptive small businesses. Although first generation cigarette-like (cigalike or G1) devices are still being marketed, the technology has since advanced to second (G2) and third generation (G3) devices featuring a user refillable e-juice tanks, high capacity batteries, and adjustable power settings. G2 and G3 devices are capable of delivering much more power than cigalike ECs, especially when coupled with a low-resistance coil. For example, a device with a 1.5 ohm (Ω) coil and 4.2 volts direct current (VDC) battery is expected to deliver 11.8 watts (W) compared to ~4 W for an older cigalike device (2.5 Ω, 3.2 VDC). These newer devices were also observed to deliver higher plasma nicotine levels than G1 devices[3]. Recently, the JUUL EC has skyrocketed in popularity (insert JUUL citation here), especially with youth. This device has led some to seek a new classification such as fourth generation (G4) since it resembles a USB memory stick more than a cigarette, but it is actually quite similar to G1 ECs in operation. JUUL uses a low powered, fixed-voltage battery with only a user-replaceable cartridge/ mouth piece. Aside from appearance and use of high concentration nicotine salt solution (~5% or 50 mg/mL) the JUUL is best classified as a successful re-boot of the G1 device class.

From basic thermodynamic principles it can be deduced that more power applied to the atomizer will produce greater vaporization because more energy is available to overcome the heat of vaporization of the e-juice. However, as the wick approaches its maximum flow rate, the energy loss from e-juice evaporation becomes limited and any further power applied to the heating coil will result in higher coil temperature but not necessairly more vaporization. Therefore the physical design of an EC atomizer and its operating power can be expected to influence many factors such as temperature, chemical profile and concentration of EC aerosol. Indeed, ECs have been shown to reach high coil temperatures. One study measured a dry coil temperature at 350°C[4], but another measured wetted coil temperatures from 139–231°C [5], and many G3 EC batteries now offer temperature control features in the range of 100–315°C.[6] Propylene glycol (PG) and vegetable glycerin (VG) are known to pyrolyze in air at high temperature[7, 8], though it is uncertain if these coil temperatures are sufficient to induce pyrolysis of PG and VG during vaporization. To this point, several researchers have found aldehydes and other carbonyl compounds in EC aerosol,[9–14] but pyrolysis of PG and VG was best demonstrated to occur during vaporization by Herrington and Myers[12]. Similarly, Kosmider et al[9] and Sleiman et al[13] found increasing levels of formaldehyde and acetaldehyde in EC aerosol of PG, VG, and PG:VG blends at voltages ranging from 3.2 to 4.8 VDC (approximately 4–10 W).

Higher vaping power increases the mass vaporized by the atomizer which subsequently condenses to form the vaping aerosol. More mass vaporized translates into a higher mass concentration of EC aerosol but the particle size distribution of said aerosol has yet to be fully characterized. Aerosol theory informs that vapor phase e-juice will begin to condense when the atmosphere is super saturated, forming nucleation centers and also condensing on ambient particles inhaled during puffing.[15] Condensation begins immediately after the vapor leaves the hot zone surrounding the atomizer heating element because of the level of supersaturation of e-juice vapor.

Calculating the vapor pressure from Antoine equation constants[16] and weighting for mole fraction, VG achieves favorable saturation ratio for self-nucleation (>4) with as little as 5.5°C increase over ambient. At 100°C vaporization temperature and 37°C inhaled oral conditions, the saturation ratio from a e-juice solution of PG, VG, nicotine and water with mole fractions of 0.29/0.65/0.05/0.01, respectively, would be 12.5 (PG), 32,700 (VG), 4.0 (nicotine), and 1.16 (water). Clearly, VG has a saturation ratio that is sufficient to initiate ultrafine particle nucleation and continue to drive condensation growth as the EC vapor cools in the mouthpiece and oral cavity. As self-nucleated condensation commences, particle count concentration becomes very large very rapidly (10^9^−10^10^) which causes particles to bump into each other (coagulation) and become larger in diameter. Due to the extremely favorable saturation ratios and high count concentrations, particles continue to grow through condensation even while they coagulate. As larger particles form (or are introduced through puff air), they act as scavengers for the very small diffusion driven particles (<100 nm) reducing the count concentration of small particles and increasing the mass of large particles which slowly increases their diameter. Nucleated condensation and coagulation was characterized by Mikheev et al[17] where ECs produced bimodal particle size distributions (PSDs) at 12 and 135 nm (cigalike model) which started unimodal for the first 2 seconds at 12 nm then shifted to bimodal at 12 and 135 nm after 2 seconds. The mass volatilized is low while atomizer temperature is low, particles form but do not grow large; as the atomizer reaches a higher steady state temperature more mass is volatilized and a second mode emerges. Mickeev et al also evaluated a higher powered G2 EC (similar to that used in this study) which produced a bimodal PSD at 20 and 170 nm. Mikheev et al recognizes the likelihood of particle evaporation due to the low pressure of the instrument used in that study, but showed dilution had minimal effect on PSD up to 26,500x dilution.

Particle size affects respiratory tract deposition [15, 18, 19], dose to tissues, potential toxicity and environmental behavior. A study by Brown and Cheng [20] identifies the lack of information on the relationships between EC power and aerosol physicochemical properties as a “critical information gap” that needs to be addressed in order to evaluate EC safety and effectiveness. To date one study has helped to fill this gap by measuring EC aerosol from 1–40,000 nm[21]. Ji et al. impinged EC aerosol into a liquid and measured the hydrodynamic diameter by dynamic light scattering. Ji et al. found a quadramodal PSD with modes around 40, 300, 2,000 and 30,000 nm, unfortunately no discussion was provided regarding potential osmotic growth of suspended EC aerosol, dissolution of EC aerosol into the water based capture solutions, nor relating hydrodynamic diameter to aerodynamic diameter. The present study specifically fills this critical information gap by characterizing EC particle size distribution in air across a broad range of sizes (16–20,000 nm), across vaping powers typical of G2 EC devices and using a mouth puff simulation.

Mouth puffing is a two-step process of using the oral cavity to draw a puff into the mouth at a low flow rate, then to inhale the puff from the mouth as a bolus at a high flow rate. [22–25]Mouth puffing provides time for the high concentration aerosol to grow by coagulation and potentially begin depositing in the oral cavity before inhalation. Measurement of EC aerosol particle size distribution (PSD) directly from the mouthpiece of an ecigarette with instantaneous dilution does not simulate the conditions of mouth puffing an EC which is performed by most users of G2 EC and most closed system devices such as JUUL.

## Methods and materials

### Electronic cigarette and puffing set-up

Vaping aerosol was produced using a G2 tank-style EC atomizer (KangerTech Protank V1, Shenzhen, China) coupled to a variable voltage EC battery or a laboratory power supply. The specific EC battery had 1300 milliampere-hour (mAh) capacity with a nominal voltage range of 3.3 to 4.8 VDC (Vision Spinner, Vision High-Technology Co., Ltd., DongGuan City, China). Using a multimeter, the actual battery voltage was measured across the range of dial settings, and EC aerosol was produced at the minimum (V_min_) and maximum (V_max_) dial settings. Using a laboratory power supply, the same EC atomizer was supplied voltage across a range broader than the battery was able to provide (3.0–6.0 VDC), with current measured during vaping. From applied voltage (V, in VDC) and measured current (I, in amperes), atomizer resistance (R, in ohms, Ω) was calculated using Ohms’ Law (V = IR). Power provided by each battery setting was calculated from measured battery voltage and atomizer resistance (P = V^2^/R). The atomizer’s e-juice vaporization rate was measured across 3.0 to 6.0 VDC in small increments. A 510 EC hub was modified to facilitate powering the atomizer directly with the laboratory power supply. e-Juice vaporization was measured by weight difference after a series of 10 puffs of 3 second duration at 20 mL/sec puff flow rate, on a 30 second puff cycle to simulate real-world puffing which allows cooling[25–27]. The Cooperation Centre for Scientific Research Relative to Tobacco (CORESTA) method 81 [27] recommends 3.0 sec puff duration and 55 mL puff volume. Since these experiments were conducted manually using a 100 mL syringe marked at 20 mL intervals, a 20 mL/sec rate was selected for convenience. The first puff was not discarded since real users do not discard their first puff. The EC tank was filled with cinnamon flavored e-juice labeled as 24 mg/mL nicotine and was estimated to be 1/3 VG, 2/3 PG, based on fluid density. This was the only e-juice evaluated in this study. All experiments were conducted in the Hudson College of Public Health, in a research lab, in a chemical fume hood. Lab make up air is supplied from the building and was used as aerosol dilution air for the first syringe dilution described below. All other dilution air was HEPA filtered compressed lab air.

### Aerosol dilution and size measurement

**Fig 1:**
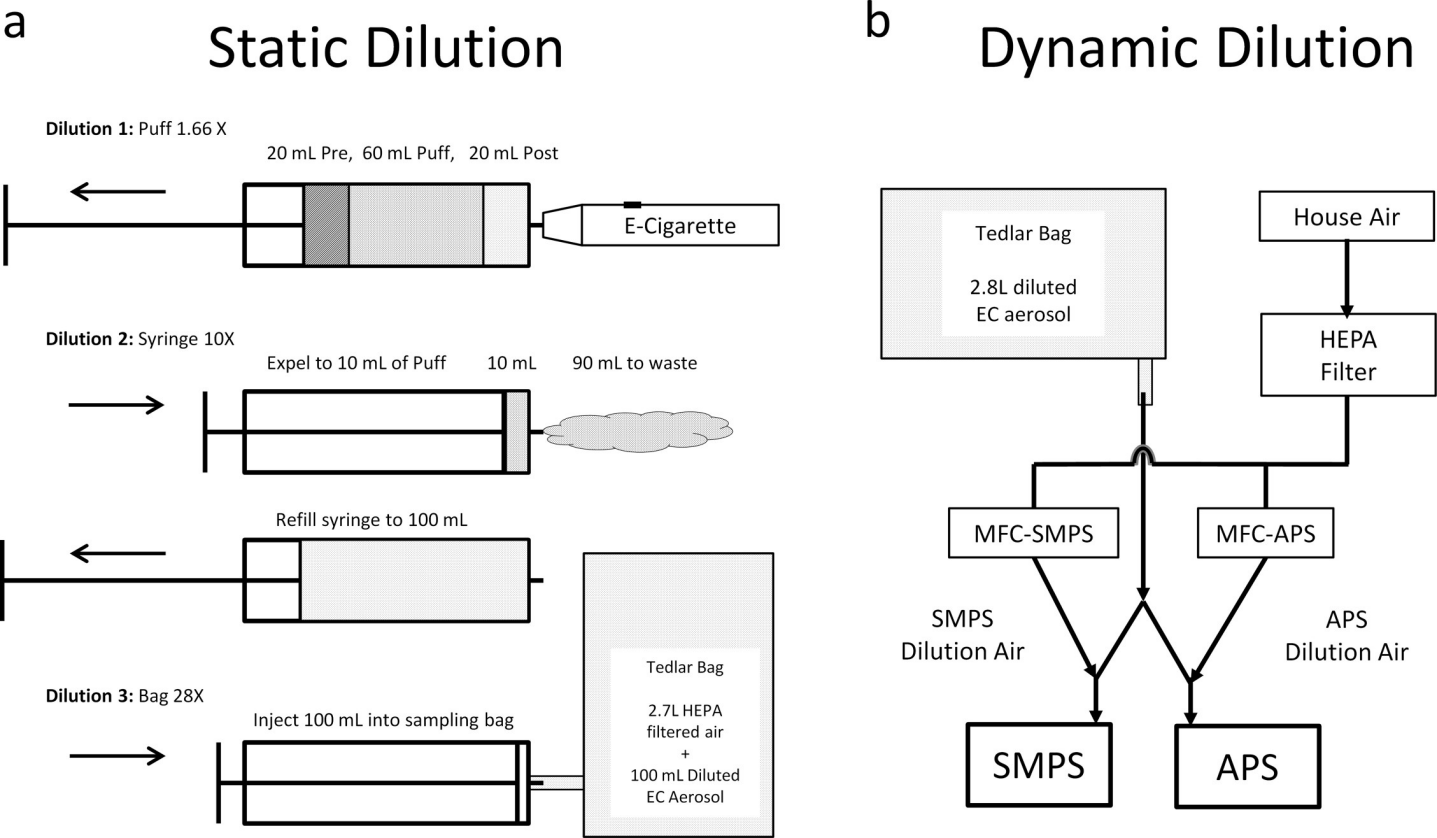
Experimental setup for diluting and sampling EC and cigarette aerosol. a.) Static dilution process of conducting a 60mL puff into a 100mL glass syringe, conducting a 1:10 syringe dilution then 1:28 dilution by injecting into a Tedlar bag for aerosol sampling. b.) System for dynamic dilution and characterization of aerosol by SMPS and APS simultaneously. Unique dilution rates were applied to SMPS and APS to achieve aerosol concentrations within single particle counting ranges. MFC—mass flow controller, HEPA—high efficiency particulate air.

As is the case with traditional cigarette smoke, vaping aerosols are extremely concentrated and must be diluted prior to measurement with real-time analytical instrumentation. Fig 1 illustrates the system that was developed to dilute the aerosol. Many combinations of static dilution and dynamic dilution were explored leading to similar particle size modes, but the relative size of each mode was affected by time to first dilution and total dilution (S1 Fig). All dilution techniques yielded a bimodal distribution in the sub 600 nanometer (nm) range, which was used to screen the dilution effects. At the lowest EC voltage (V-min) the simple 1:26 bag dilution allowed the greatest time for the primary puff to coagulate at high concentration while the syringe was being connected to the dilution bag and had the largest ratio of large to small particles. The 1:26 bag + 1:6 dynamic dilution showed similar shaped PSD but with evaporation effects causing a left shift in particle modes and reduction in concentration. The 1:10 syringe dilution + 1:26 bag dilution yielded a more balanced distribution of size modes indicating prevention of coagulation more so than evaporation since the 300 nm mode was roughly the same size as the bag+dynamic dilution, but the 50 nm mode was larger. The 1:100 syringe dilution + 1:26 bag dilution yielded similar particle modes as the 1:10 syringe + 1:26 bag dilution but with more noise in the plot due to the larger dilution correction. From these results we concluded that, a longer time to first dilution appeared to produce more of the larger particles and fewer small particles, due to coagulation and the scavenging effect of larger particles on small particles. Screening trials conducted at the higher power condition (V-max) showed a distinct right shifting of all distributions and a bias toward the larger mode which further confirmed the need to quench coagulation as soon as the puff was completed. The noise observed in the 1:100 syringe + 1:26 bag dilution was deemed excessive and not worth the added uncertainty for the minimal effect it showed on PSD.

Based on these results a 10x syringe dilution, followed by 28x bag dilution (for aerosol storage), followed by independent dynamic dilution specific for each instrument was chosen (Fig 1). The chosen dilution technique was primarily focused on quickly reducing particle count concentrations to levels that would quench coagulation effects and produce particle number concentrations within the single particle counting range of each instrument after dynamic dilution. Particle counts are stable for several minutes if below 10^7^ particles / cm^3^. [15] From initial experiments and searching the literature we expected particle counts of 10^8^−10^9^ particles/ cm^3^ in the primary aerosol, so we anticipated a 100x dilution would be necessary to quench coagulation. By “freezing” the particle size distribution after the mouth puff simulation, the aerosol PSDs obtained should represent particles that enter the respiratory tract when the mouth puff is inhaled. To reduce EC aerosol evaporation within the bag, the interior of the bag was coated with e-juice to pre-saturate the air with e-juice vapor. This was accomplished by allowing several EC puffs to deposit on the side walls until a noticeable film developed. When evacuating the bag before a trial it would appear to be wet inside.

A standardized puff was conducted as follows: using a 100 mL glass syringe, a 60 mL puff was conducted over a 3-second period with 20 mL preceding the puff to establish steady flow and 20 mL following puff to clear aerosol from the tubing for a total volume of 100 mL and dilution factor of 1.67x. Of this, 90 mL was immediately expelled to waste and the plunger re-filled to 100 mL to provide an additional 10x dilution. This 100 mL of diluted aerosol was injected into a sampling bag pre-filled with 2.7 L of HEPA filtered air. The bag was briefly massaged to mix the aerosol. The combined trifold dilution of puff-syringe-bag was 467x. Aerosol was sampled from the bag through ¼ inch (6.35 mm) inside diameter conductive tubing and split into parallel streams for simultaneous analysis by a Scanning Mobility Particle Sizer (SMPS) and an Aerodynamic Particle Sizer (APS) (both from TSI, Inc., Shoreview, MN). Dynamic dilution factors were 9.6x for the SMPS and 32x for the APS, respectively, yielding a total dilution factor of 4,480x for the SMPS and 14,933x for the APS. For example: the APS samples at 5 L/m, to achieve a 32x dilution 4.84 L/min of dilution air was supplied through a mass flow controller with the balance of (0.16 L/min) drawn from the bag of test aerosol. Total tubing length to SMPS and APS was 71.1 cm and 61.0 cm, respectively. Tubing loss calculations were estimated for each size bin and found to be 5–9% for particles 16–25 nm, 1–5% for particles 25–100 nm, and <1% for particles larger than 100 nm. See S1 Table for size bin specific loss estimates. Using this system of static dilution into a sampling bag plus independent dynamic dilution for each aerosol instrument allowed measurements to be conducted with a minimally changing aerosol (S2 Fig) source that could be sampled without further diluting the source. This is not possible using a rigid container such as a glass carboy or exposure chamber.

The total time to conduct puff (3 sec), dilute with syringe (1 sec), and inject into the bag (3 sec) was approximately 7 seconds. The first sample analysis was initiated after 30 seconds to allow aerosol within the bag to fully mix, to connect the bag to the instrumentation tubing, and flush the tubing volume with aerosol. After puff-syringe-bag dilution the aerosol size distribution is stable and changed minimally over time as illustrated in S2 Fig. The SMPS, as configured for this work, measured particles from 16 to 583 nm over a 45-second scanning period with 15 second retrace. The APS measured particles from 514 nm to 19.8 μm size range over a 50-second sample averaging period with 10 second pause so that both instruments gave an output every 60 seconds. Thus, the total particle size measurement range was 16 nm to 19,800 nm (0.016 to 19.8 μm). Our search of the literature indicates this is the first study to use these instruments together to measure vaping aerosol.

For comparison purposes, a commercially available filtered cigarette (Kool Blue Menthol, R.J. Reynolds Tobacco Company, Winston-Salem, NC) was puffed, diluted and characterized using the same technique as the EC aerosol.

Key differences in this experiment and others[4, 12, 17, 21, 28, 29] is the 3 second puff into a glass syringe which simulates mouth puffing and using a combination of static and dynamic dilutions into a bag (rather than a rigid container) to stabilize the aerosol and allow continuous sampling without diluting the aerosol.

## Results

### EC battery performance and vaporization per puff

Voltage delivered by the EC battery differed considerably from the voltages indicated on the dial, varying linearly from 3.15 VDC at the minimum setting (3.3 VDC) to 5.83 VDC at the maximum setting (4.8 VDC) following a linear regression of V_actual_ = 1.89V_indicated_—3.28, R^2^ = 0.9999. Using Ohms Law and measurements of actual battery voltage at V_min_ and V_max_, the expected power at V_min_ is 3.3 W and V_max_ is 10.5 W. When powered by the laboratory power supply from 3.0 to 6.0 VDC, the atomizer current increased linearly, indicating a constant atomizer resistance of 3.02 ± 0.019 Ω (mean ± standard deviation). Therefore, the vaping power ranged from 3.0 W at 3.0 VDC to 11.9 W at 6.0 VDC.

**Fig 2:**
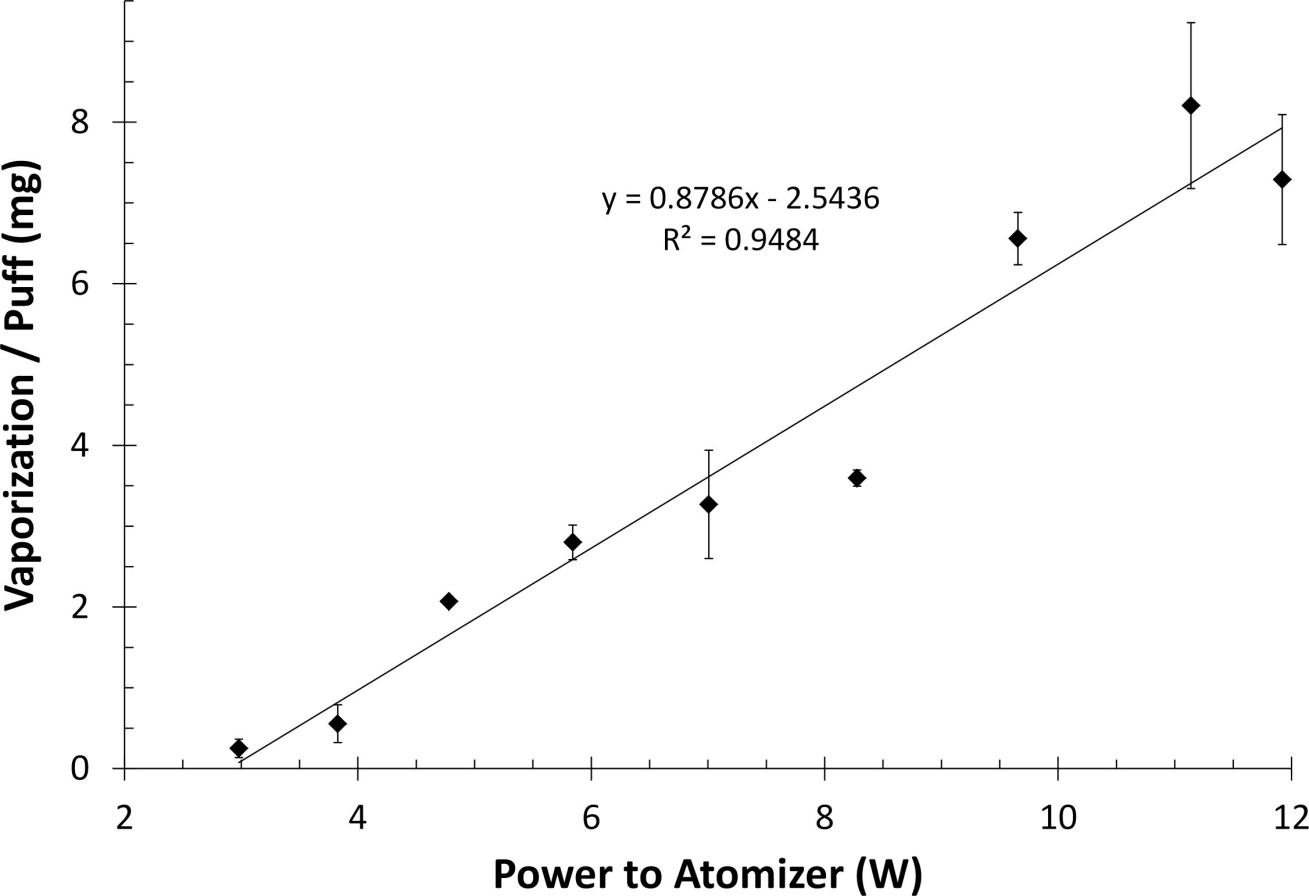
Vaporization rate per puff of a G2 EC atomizer with total resistance of 3.02Ω. Vaporization rate per puff of a 3.02Ω single coil EC atomizer was measured by weight difference across a 10 puff series with 30 seconds between puffs at 9 wattages. EC atomizer was powered by a laboratory power supply for these experiments. Each point is the average vaporization per puff with standard error bars.

Fig 2 shows the e-juice vaporization during the 10-puff series increased linearly with power from 3.0 W (3.0 VDC) to 11.9 W (6.0 VDC) indicating an 86x increase in mass vaporized per puff for a 4x increase in atomizer power (doubling of atomizer voltage). This figure also shows that trial replicates had good reproducibility even though performed manually; however there seems to be a systematic negative bias around 9 W for this device that is not due to experimental variability. This was also observed in the PSD trials below where the 9 W trial had lower values than expected.

### Aerosol characteristics

**Fig 3:**
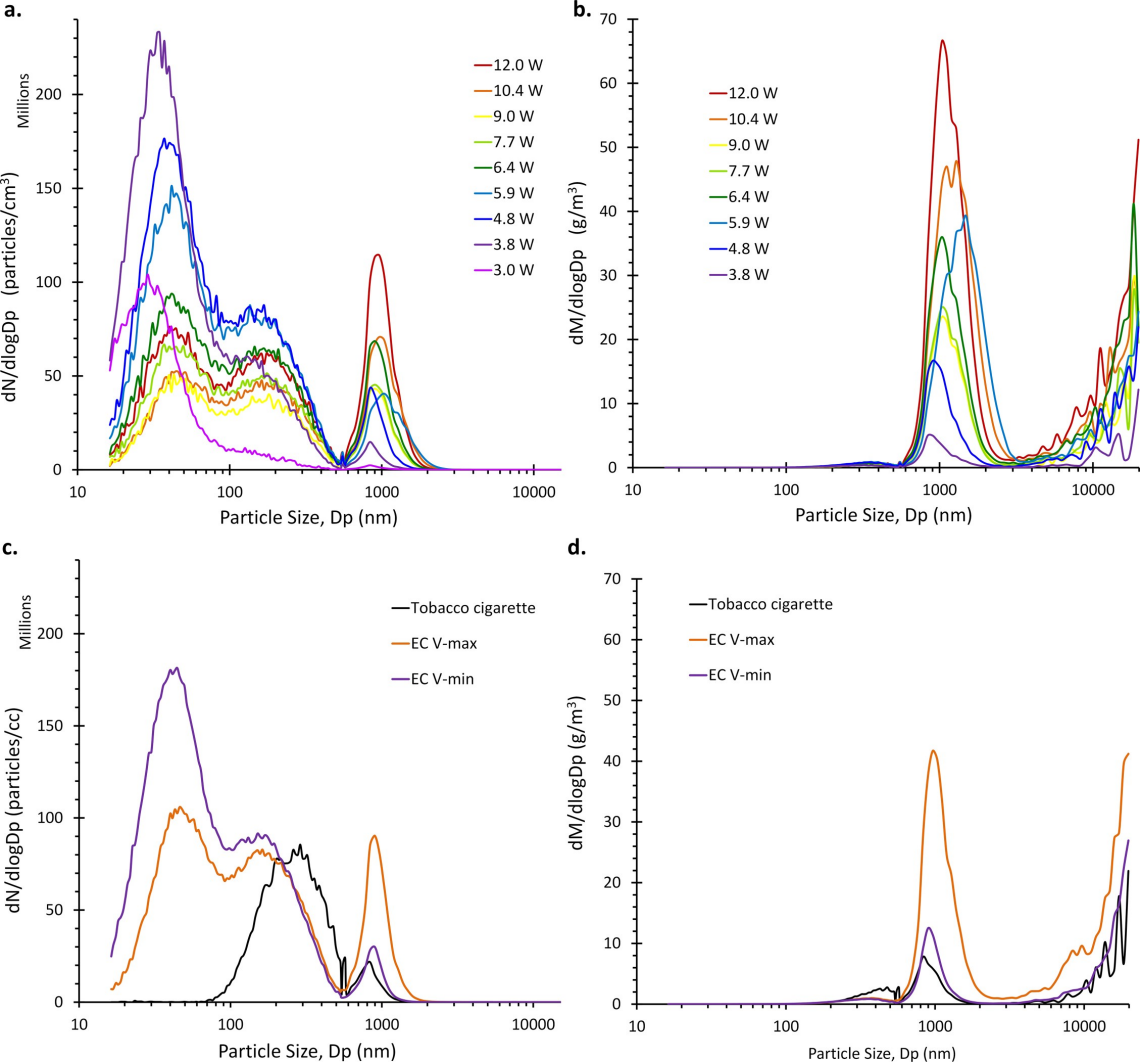
Particle size and mass distributions for EC aerosols and tobacco smoke. a.) EC aerosols produced by a 2^nd^ generation EC atomizer powered by a laboratory power supply at 9 different levels. Nano-sized particle count decreased as power increased but micron sized particles increased as power increased. b.) Mass distribution of EC aerosol calculated from particle size distribution. Large particles make up the majority of aerosol mass. c.) EC aerosols produced by the same atomizer powered by a variable voltage EC battery at V_min_ and V_max_ dial settings and compared to a Kool Blue cigarette smoke aerosol. Note the distinct difference between EC aerosol and cigarette smoke. Data shown are average of three trials. d.) Mass distribution of EC aerosol and cigarette smoke calculated from particle size distribution. Battery powered EC aerosol has a smaller particle mode around 1 μm than observed in power supply experiments which may be due to battery underperformance. Data shown are average of three trials.

The particle size distribution (PSD) measured by the combined SMPS/APS system indicated a trimodal aerosol with two modes in the measurement range of the SMPS at 40 and 200 nm and one mode in the APS measurement range at ~1000 nm, see Fig 3A and 3C. A fourth mode may be present above 20,000 nm, but is only visible when looking at the mass distribution (Fig 3B and 3D) and is quite noisy due to the relatively few particles of high mass at this large diameter. Overall, the trend is quite clear; as atomizer power increased the number of nanosized particles decreased but the number of micron sized particles increased. Fig 3C shows the aerosol particle size distribution at the minimum and maximum battery settings. V_min_ corresponds well with the 3.8 W curve obtained with the power supply, and V_max_ corresponds well with the 10.4 W curve from the power supply. Overall the mass distribution shows lower output by the battery driven atomizer, which is most easily observed at the 1000 nm peak in Fig 3B and 3D. As noted above, the 9 W PSD values are lower than expected at all levels which is suspected to be unique to the test device and not variability between trials or something expected to occur on a different device. It also appear that the nano-sized particle count is increasing at 12W.

Cigarette smoke was found to have a bimodal distribution with the dominant count mode at ~300 nm similar to other’s findings[19, 30, 31] and secondary mode at ~800 nm (Fig 3C) similar to that found in Sahu et al. [19] for exhaled cigarette smoke.

Mass distributions were calculated from SMPS and APS count data assuming spherical particles with specific gravity of 1.10 for EC aerosol and 1.18 for tobacco smoke and accounting for dilution factors. Fig 3B indicates a prominent mass peak at ~1000 nm for the EC aerosol with a potential second mass peak beyond the measurement range of 20 μm, which supports findings in Ji et. al[21] through dynamic light scattering of liquid impinged EC aerosol. In Fig 3B and 3D it is quite obvious that aerosol mass is dominated by particles greater than 600 nm which has been the upper limit of many other EC aerosol studies [4, 17, 28, 29]. As shown in Fig 3C and 3D, the tobacco cigarette count distribution was substantially different from the two e-cigarette count distributions in that there was only one mode in the sub-micrometer size range, at approximately 300 nm. The e-cigarette count size distributions at both the lower and higher voltages had two modes in that range, at approximately 40 and 200 nm. However, the mass distributions were similar, with a mode at 1 μm.

**Fig 4:**
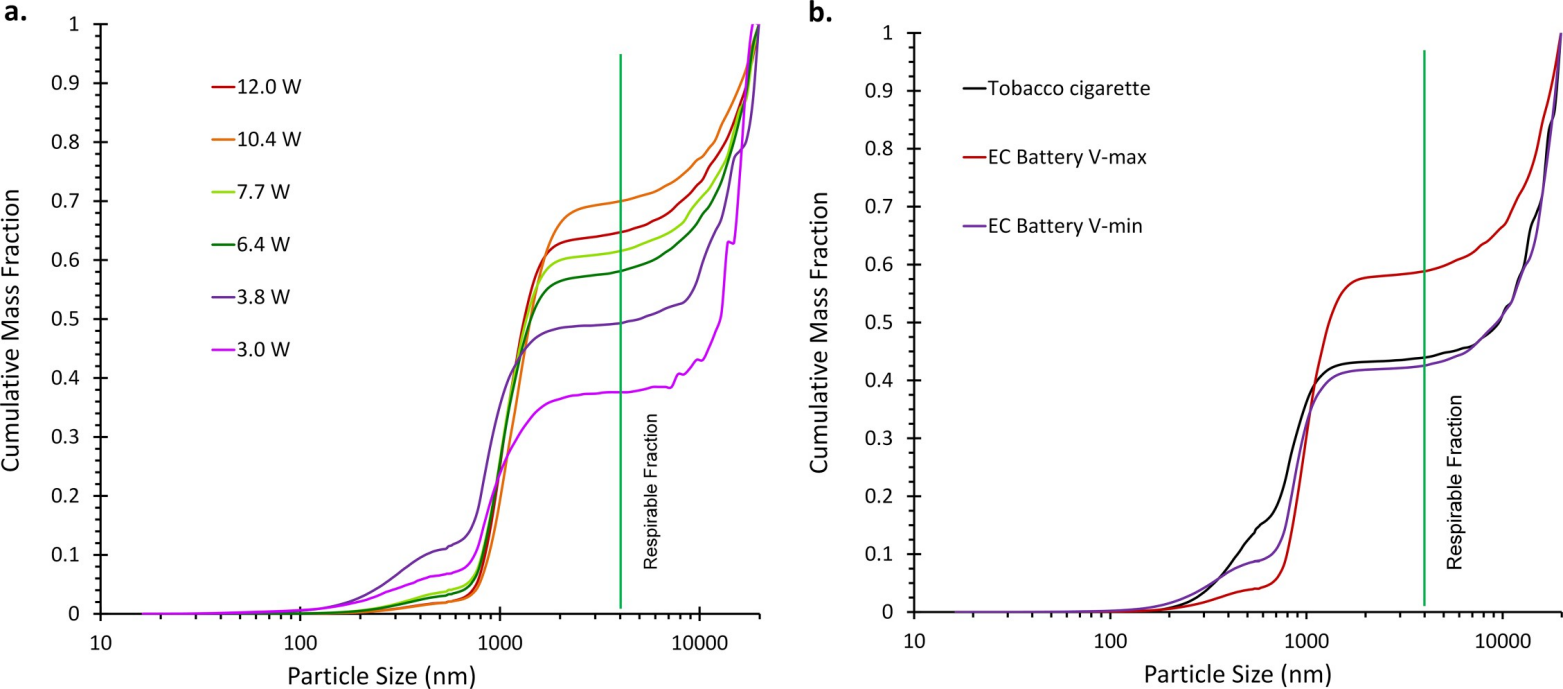
Cumulative mass fraction by size for EC aerosol and cigarette smoke. a.) EC aerosol generated with laboratory power supply at 9 levels. As power increases, a greater fraction of the mass is below 4 μm which is within the respirable fraction, left of the vertical line. b.) Cigarette smoke (Kool Blue) and EC aerosol powered by a variable voltage EC battery at V_min_ and V_max_ dial settings. Respirable fraction of cigarette smoke and EC aerosol at minimum dial setting are very similar. Respirable fraction does not increase as greatly when powered by battery compared to laboratory power supply, this may be due to battery underperformance.

Cumulative mass distributions are presented in Fig 4 and show that as power increased, cumulative mass fraction shifted left, into the respirable (alveolic) region (<4 um) due to the growing mass of the ~1000 nm peak. This large portion of aerosol mass would have been overlooked if using the SMPS alone. The respirable mass fraction increased from 37% at low power to 69% at high power. The percentage of total mass found in the ~1000 nm peak alone increased from 27% at low power (3.0W) to 65% at high power (11.9W). The cigarette smoke aerosol had a greater fraction of mass <600 nm than did most EC trials, but the respirable fraction was very similar to lower powered EC trails.

## Discussion

### Operational limitations of the EC atomizer and battery

During optomization experiments, the atomizer was evaluated as high as 14W (6.5VDC) but failed after a few puffs, which led us to use 11.9 W (6.0VDC) as the upper limit. The cause of failure is unknown, but is suspected to be melting of the solder joint between the non-resistive leads and resistive coil. Coil temperature was not measured in this work, but Schripp et al.[4] thermographically measured a dry coil of a similar EC atomizer at 350°C and Zhao et al.[5] measured wet coil temperatures of four cig-a-like ECs from 139–231°C. It is also noteworthy that the battery used in this study had considerable inaccuracy but in our experience newer models of similar batteries are much more accurate.

### Particle size distribution

The particle size distribution of aerosols <600 nm in this study are in good agreement with Schripp et al.[4], who reported bimodal aerosol with modes at ~45 and 200 nm for a PG-based aerosol aged 1 minute in a 10-L glass chamber, measured with a Fast Mobility Particle Sizer (FMPS). Our data are also consistent with observations of Zhang et al.[18], who reported modes at ~100 and 370 nm for dynamically diluted PG aerosol measured by SMPS. The particle size distribution at 3.0W observed in this study was unimodal around 30 nm which is similar to the unimodal (25 nm) distribution measured by Zhao et al.[5] using SMPS and is similar to other low powered cigalike ECs[21, 29].

As power was increased more e-juice was vaporized and available to form a greater number of particles and grow those particles by condensation. Mouth puffing fosters particle growth by coagulation and is mostly responsible for the 1000 nm mode. With increasing power, more particles are formed which causes faster coagulation which grows the 1000 nm mode. Due to particle scavenging, the 1000 nm particles act as a sink for small particles that are driven by brownian motion which reduces the nano-sized counts even as they are forming. Above 10 W, it appears that the nano-sized particles are beginning to increase in count concentration, but this is uncertain since these experiments were only performed once and this could be due to measurement variability. All this together indicates that at low power a G2 EC will perform similar to a cigalike (G1) EC, but as power is increased the PSD shifts, and ~1000 nm particle mode emerges to dominate the mass distribution. There appears to be a 4^th^ mode emerging above 20 μm but this is uncertain due to the noise in this measurement range and limitations of the instrumentation used in this study.

Other EC aerosol studies [17, 28, 29] have suggested that aerosol dilution and operating pressure greatly affect the PSD of EC aerosol due to particles evaporation. Mikheev et al.[17] measured the particle size distribution of EC aerosol using a differential mobility spectrometer (DMS) which operates at a substantial vacuum and found modes at 12 and 130 nm at low dilution (1:30) and 17 and 80 nm at high dilution (1:26,500), suggesting a limited role in evaporation in fast analyzers such as DMS. In S1 Fig we observed a left shifting of the particle size distribution as 1:6 dynamic dilution was added to the 1:26 bag dilution, this is clearly an evaporation phenomenon which resulted in lower counts and smaller particles. Smaller particle sizes observed from the syringe+bag dilution are. Diluted aerosol will have fewer particle-to-particle collisions than highly concentrated aerosols and will result in modest particle growth over the minute time-scale that was needed to complete these experiments, S2 Fig illustrated how our aerosol changed over time in the nano-size range. To minimize evaporation from our bag dilution step inside of the bag was coated with e-juice and pre-filled with dilution air several minutes before the experiment. This allowed the dilution air to saturate with each of the e-juice components so that the injected puff was not further evaporated within the bag. Since we needed to use dynamic dilution to further reduce our aerosol concentration we expect the PSDs reported in this study to be an underestimation of the true PSD sampled which does not diminish the importance of the results observed in this study.

Second to vaporized mass, inhalation style of the EC user is probably the most critical factor in determining the amount of agglomeration that EC aerosol will experience since this will determine the duration the aerosol spends at high particle concentration. A mouth puff remains minimally diluted for the duration of the puff before inhalation transports it to the lungs as a plume.[32] Our syringe puff technique with extra volume in the syringe is similar to mouth puffing and the injection into the bag is similar to inhaling the puff into the lungs. Although primary particles formed during vaping are likely very small[17], they rapidly age while in the mouth and agglomerate to form a heterogeneous aerosol as shone in this study and others[23, 25]. Realistic simulation of EC puffing is needed to predict the effects of device settings and puff style on aerosol characteristics and ultimately physiological impacts such as respiratory tract deposition.

### Shift in the respirable fraction with increasing power

In contrast to studies that only investigated airborne particles smaller than ~600 nm[5, 12, 17, 18, 21, 28], this study investigated particles up to 20,000 nm (20 μm). In Fig 4 the cumulative mass fraction by particle diameter indicates that the vast majority of EC aerosol mass was from particles larger than 600 nm (0.8 μm). The only other study to our knowledge that has investigated beyond ~600 nm was Ji et al. [21] who used an SMPS to measure 7–289 nm airborne aerosol and dynamic light scattering (DLS) to measure liquid impinged EC aerosol from 1–40,000 nm. The DLS technique determined the PSD to have four modes at 25, 200, 900 and 23000 nm. In the present study, three modes are clearly seen in Fig 3B and 3D, and possibly the leading tail of a 4^th^ mode beyond the measurement range of our instrumentation.

Based on this laboratory simulation, a 10-puff session would result in 2.5–72.5 mg e-juice inhaled, with 37–69% of aerosol being < 4 μm in size and highly respirable. For e-juice containing 24 mg/mL nicotine, this would be an intake of 0.09–1.74 mg nicotine. The observed shift in vaping aerosol mass distribution toward larger particle sizes from higher power settings was likely due to greater mass of e-juice vaporized, resulting in a greater saturation ratio and more vapor mass available to form primary particles and grow particles. This increased the number of primary particles which increased the coagulation rate which led to rapid particle growth and count reduction among the nano-sized particles while inside the puffing syringe (mouth simulation). After syringe and bag dilution, the aerosol particle sizes were quite stable as shown in S2 Fig.

As Ingebrethsen et al.[28] and Fuoco et al.[29] have suggested and we observed in our optomization experiments, high levels of dilution during aerosol measurement causees EC aerosol evaporation which biases size estimates towards smaller values, thus causing an underestimation of mass distributions. Even in the face of this possibility, our data clearly demonstrate that a substantial fraction (~95%) of the measured aerosol is well above the ranges (10–600 nm) previously measured and reported. Aerosol particle size is well known to determine the efficiency and location of particle deposition along the respiratory tract [33–35]. Based on current models of particle deposition[32, 36, 37], the majority of smaller EC particles are expected to deposit in the alveoli, but the dominant EC mass mode (1–2 μm) will deposit in the oral and pharyngeal regions at nearly twice the rate of alveolar deposition. These data are highly relevant for the investigation of potential toxicity to tissues chronically exposed to EC aerosol. Size selective pre-sampling for large diameter particles should be conducted to verify the presence of a particle mode at or above 20 μm.

### Limitations

This study has many limitations which should be noted and briefly discussed. First this study only examined a single 2G EC with a single e-juice out of hundreds of options currently on the market. While it was the objective of this study to observe trends that should be universal to all similar devices, each device and e-juice combination is expected to have unique results. The puff regime simulated a “typical” vaping scenario that was very similar to the CORESTA recommended method number 81 for evaluation of electronic cigarettes but the literature shows puff topographies vary across users and across devices. Lower puff rate and longer puff duration are expected to result in larger PSDs since the vaporized mass will be less diluted and in closer proximity, but these factors were not assessed in this study. The dilution technique utilized was not ideal but was the best that could be accomplished with the available resources at the time of conducting these experiments. Conducting puffs into a syringe provided a simple way to simulate one aspect of mouth puffing, but not physiological temperature and relative humidity which are both expected to cause further particle growth by condensation. Manually conducting puffs introduced human error into the reproducibility of the results which is why triplicate trials were averaged for the battery powered EC trials and why the lab powered EC trials were conducted over a wide range of powers to show a trend not to make a statistical test. The trend is quite apparent that increasing power decreased the nano-sized particles and increased the micron sized particles systematically.

## Conclusions

Based upon the results of the specificly tested EC device, with a single e-juice, puffed at a single flow rate using a simulated mouth puff; vaping aerosol is dynamic and mouth puffed EC aerosol spans a much wider particle size range than previously reported. Although the major portion of particle mass is still well within the respirable size range, it is near the upper bound which shifts the primary deposition site to the oro-pharyngeal region instead of the alveoli. These results demonstrate the dramatic effect increasing power has on EC aerosol particle concentration and mass distribution across a wide range of particle sizes. Because e-cigarette technology has continued to evolve toward higher power devices, and the mass of vaporized e-juice increases with power, there is a great need for further research to inform the design and regulation of e-cigarette products and critical components such as batteries, atomizers and e-juice composition to ensure a safe and reliable vaping experience.

## Supporting information

S1 Fig Effects of dilution techniques and dilution factors on particle size distribution using a combination of static dilutions or static+dynamic dilution. The primary consideration of these trials were to establish a dilution technique that could be uniformly applied to all test conditions to create a minimally changing aerosol that was dilute enough to measure within the instrument single particle counting range with minimal noise due to dilution correction. EC aerosol generated by EC battery at a.) minimum dial setting and b.) maximum dial setting were used for these experiments. Dilution with static (bag) and dynamic technique presented less noise than static techniques alone. Particle size distribution is left shifted with bag+dynamic dilution which is due to evaporation and prevention of particle agglomeration.
(TIF)

S2 Fig Ageing of EC aerosol diluted using 1:10 syringe + 1:27 bag to determine the effect on particle size distribution. a.) EC aerosol generated with EC battery at minimum dial setting and b.) maximum dial setting. Ageing causes right shifting in the nanosized particles at minimum and maximum battery voltage but minimal change in the larger mode.
(TIF)

S1 Table Tubing loss calculation for each size bin within the SMPS/APS ensemble range. (TIF)
