# Supplementary figures and images for: Electronic cigarette power affects count concentration and particle size distribution of vaping aerosol
Source: PLoS One. 2018 Dec 31;13(12):e0210147. doi: 10.1371/journal.pone.0210147 (PMC6312322; doi:10.1371/journal.pone.0210147)

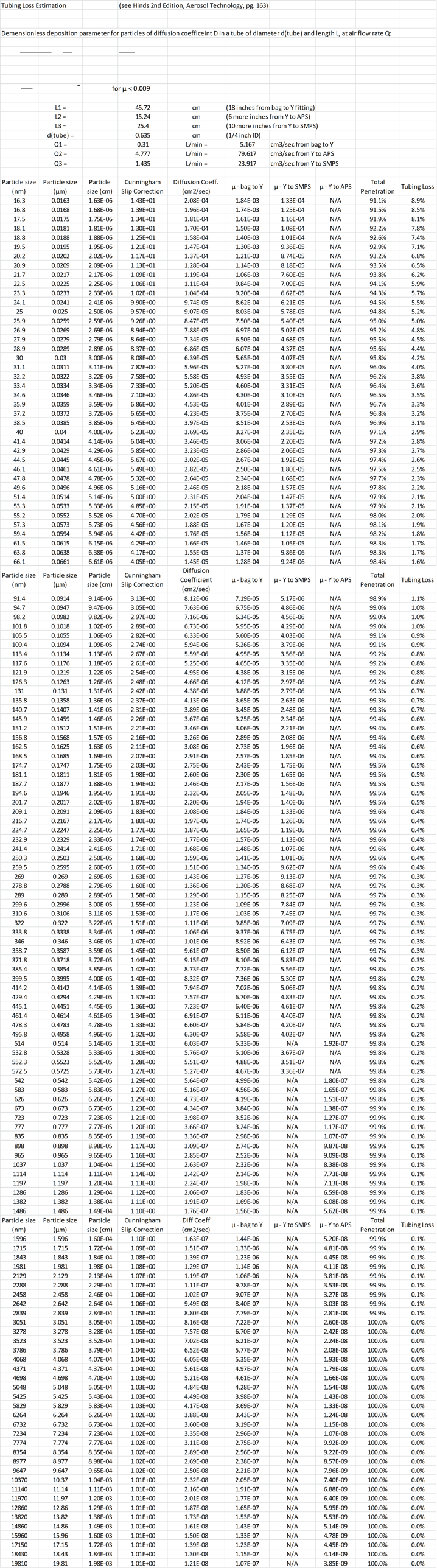

Supplement: S1 Table — (TIF) [file pone.0210147.s003.tif]
